# Brain volumes, cognitive, and adaptive skills in school-age children with Down syndrome

**DOI:** 10.1186/s11689-024-09581-6

**Published:** 2024-12-19

**Authors:** Rebecca Grzadzinski, Kattia Mata, Ambika S. Bhatt, Alapika Jatkar, Dea Garic, Mark D. Shen, Jessica B. Girault, Tanya St. John, Juhi Pandey, Lonnie Zwaigenbaum, Annette Estes, Audrey M. Shen, Stephen Dager, Robert Schultz, Kelly Botteron, Natasha Marrus, Martin Styner, Alan Evans, Sun Hyung Kim, Robert McKinstry, Guido Gerig, Joseph Piven, Heather Hazlett, for the IBIS Network, Rebecca Grzadzinski, Mark D. Shen, Jessica B. Girault, Tanya St. John, Juhi Pandey, Lonnie Zwaigenbaum, Annette Estes, Stephen Dager, Robert Schultz, Kelly Botteron, Martin Styner, Alan Evans, Robert McKinstry, Guido Gerig, Joseph Piven, Heather Hazlett, C. Chappell, D. Shaw, J. Constantino, J. Elison, J. Wolff, J. Pruett, D. L. Collins, V. Fonov, L. MacIntyre, S. Das, K. Truong, H. Volk, D. Fallin

**Affiliations:** 1 Carolina Institute for Developmental Disabilities (CIDD), University of North Carolina at Chapel Hill, 101, Renee Lynne Court, Carrboro, NC 27510 USA; 2 Department of Psychiatry, University of North Carolina at Chapel Hill, Chapel Hill, NC USA; 3 New England College of Optometry, Boston, MA USA; 4 University of Washington Autism Research Center, Seattle, WA USA; 5 Department of Speech and Hearing Sciences, University of Washington, Seattle, WA USA; 6 Center for Autism Research at the Children’s Hospital of Philadelphia, Philadelphia, PA USA; 7 Autism Research Centre, Department of Pediatrics, University of Alberta, Edmonton, Canada; 8 Easter Seals, UCP, Raleigh, NC USA; 9 Center On Human Development and Disability, University of Washington, Seattle, WA USA; 10 Department of Psychiatry, Washington University in St. Louis, St. Louis, MO USA; 11 Montreal Neurological Institute, McGill University, Montreal, Canada; 12 Mallinckrodt Institute of Radiology, Washington University in St. Louis, St. Louis, MO USA; 13 Department of Computer Science and Engineering, New York University, New York, NY USA

**Keywords:** Down syndrome, Autism spectrum disorder, Neurodevelopmental disorder, Intellectual disability, Neuroimaging, Neurobehavioral/behavioral profiles, School-age children, Brain volumes, MRI, Cognitive, Adaptive, Cortical volumes

## Abstract

**Background:**

Down syndrome (DS) is the most common congenital neurodevelopmental disorder, present in about 1 in every 700 live births. Despite its prevalence, literature exploring the neurobiology underlying DS and how this neurobiology is related to behavior is limited. This study fills this gap by examining cortical volumes and behavioral correlates in school-age children with DS.

**Methods:**

School-age children (mean = 9.7 years ± 1.1) underwent comprehensive assessments, including cognitive and adaptive assessments, as well as an MRI scan without the use of sedation. Children with DS (*n* = 35) were compared to available samples of typically developing (TD; *n* = 80) and ASD children (*n* = 29). ANOVAs were conducted to compare groups on cognitive and adaptive assessments. ANCOVAs (covarying for age, sex, and total cerebral volume; TCV) compared cortical brain volumes between groups. Correlations between behavioral metrics and cortical and cerebellar volumes (separately for gray (GM) and white matter (WM)) were conducted separately by group.

**Results:**

As expected, children with DS had significantly lower cognitive skills compared to ASD and TD children. Daily Living adaptive skills were comparable between ASD children and children with DS, and both groups scored lower than TD children. Children with DS exhibited a smaller TCV compared to ASD and TD children. Additionally, when controlling for TCV, age, and sex, children with DS had significantly smaller total GM and tissue volumes. Cerebellum volumes were significantly correlated with Daily Living adaptive behaviors in the DS group only.

**Conclusions:**

Despite children with DS exhibiting lower cognitive skills and smaller brain volume overall than children with ASD, their deficits in Socialization and Daily Living adaptive skills are comparable. Differences in lobar volumes (e.g., Right Frontal GM/WM, Left Frontal WM, and Left and Right Temporal WM) were observed above and beyond overall differences in total volume. The correlation between cerebellum volumes and Daily Living adaptive behaviors in the DS group provides a novel area to explore in future research.

**Supplementary Information:**

The online version contains supplementary material available at 10.1186/s11689-024-09581-6.

Down Syndrome (DS) is the most common congenital neurodevelopmental disorder (NDDs) with a prevalence rate of 1 in 700 live births [1, 2]. DS is diagnosed when genetic testing reveals an additional copy of the 21st chromosome, referred to as Trisomy 21. Characteristics of DS include differences in facial features (e.g., flattened nasal bridge, almond-shaped eyes), body characteristics (e.g., small hands and feet, low muscle tone) and intellectual differences (mild to moderate intellectual disability) [1, 3, 4]. While DS has a known congenital cause, ongoing research is still working to understand its impact on brain development.

Existing research suggests that individuals with DS exhibit distinct neuroanatomical differences compared to typically developing children. Magnetic resonance imaging (MRI) studies have shown reductions in brain weight, cerebellum size, and atypical cortical surface features in individuals with DS, albeit with small sample sizes [1]. Studies have also shown differences such as decreased total brain volume [5–7], total gray matter (GM) and white matter (WM) volumes [8], reductions in frontal WM volume [9], increased parietal GM and WM [7, 9], increased temporal GM and WM [7, 9], as well as increased occipital GM [9]. Studies including both children and adults with DS have reported reduced WM integrity in various brain tracts, such as frontal tracts [10, 11], corpus callosum [11], and cortico-spinal tracts [10, 12]. Voxel-based morphometry MRI on 21 children and adolescents with DS (mean age = 10.5 years, SD = 3.3; age range: 7–16 years) identified GM volume reductions in the cerebellum, hippocampus, parahippocampal gyri, and the frontal lobe. Additionally, Carducci et al. (2013) found preservation of parietal and temporal GM volumes in children with DS when compared to TD children. [13] Menghini et al. [14] and Pinter, Eliez, et al. (2001a) found decreases in total GM and WM in DS studies focusing on school age children through adolescence [8, 14]. Notably, studies in this area have mainly included adult samples, with limited inclusion of children, such as Gunbey et al. (2017), which included a small group of DS toddlers (N = 10, ages 2–4 years old), [10] and two other studies that included N = 12 and N = 23 school age children with DS (Mean age = 5.94 and Mean age = 6.7) [5, 6]. This suggests that individuals with DS have unique patterns of brain development that may contribute to cognitive and functional differences observed in the population. However, the small sample sizes and broad age ranges emphasize the need for more comprehensive research to better understand these patterns in DS.

DS mouse models, such as Ts65Dn, found an overexpression of GIRK, a potassium channel that can alter excitatory input in different brain regions, such as the hippocampus and prefrontal cortex [15–17]. Other studies have reported altered DYRK1A gene expression in the cerebellum, which is necessary for brain growth in mice and humans [18, 19]. Cognition and memory research in children with DS have also revealed distinct patterns. Pennington et al. [20] investigated prefrontal (holding information or working memory) and hippocampal (episodic information in long term memory [LTM]) functions in DS. The study found that children with DS (Mean age = 14.7 years, SD = 2.7) performed worse on all hippocampal measures, including a virtual Morris water maze (*p*< 0.05), compared to typically developing children who were mental age-matched (Mean age = 4.9 years, SD = 0.75), but not on prefrontal measures (Ecological Memory Index) [20]. All hippocampal, or LTM dependent measures, were converted to z-scores, or composites, for comparison, indicating that the DS group performed significantly worse on the NEPSY List (*p* < 0.05) and Paired Associates (*p* < 0.001) than their typically developing mental age-matched counterparts. The prefrontal composites were better in DS children, but no significant group differences were found. Further research is needed to dissect the relationship between cognition and brain morphology.

This study was motivated by the limited research on the brain and behavior development in children with DS. To address this gap, a sample of school-aged children with DS completed neuroimaging and behavioral assessments, and were compared to same-aged typically developing (TD) and autism spectrum disorder (ASD) comparison groups. The overlapping behavioral characteristics between DS and ASD makes ASD a particularly important contrast group. Research estimates that approximately 10% of individuals with DS also have diagnoses of autism, rates 5–10 × higher than the general population [21, 22]. Intellectual disability is characteristic of DS and present in approximately 30–50% of ASD individuals [23, 24]. Additionally, individuals with DS share challenges with ASD individuals in verbal and social-communicative abilities [23, 25, 26], and atypical patterns of sensory reactivity [27–30], though the presentation of these behaviors is highly heterogeneous across both diagnoses. One recent study compared DS individuals with co-occurring autism (DS + ASD) to ASD individuals with intellectual disability ID (ASD + ID), and individuals with ID that did not meet criteria for ASD (ID; [26]). Results indicated that the DS + ASD and ASD + ID groups had similar ASD symptoms as well as similar cognitive scores (that were higher than those with ID alone). However, those with DS + ASD had elevated disruptive behaviors and lower adaptive functioning compared to the other groups, highlighting the unique behavioral profiles that may exist in certain co-occurring groups [26]. While the diagnoses of DS and ASD are distinct, research has identified overlaps in behavior. Comparing DS with ASD allows us to evaluate whether neurobiological patterns are specific to DS or if they overlap with those observed in ASD.

No study, to our knowledge, has 1) evaluated brain volumes in school-age children with DS, 2) directly compared the neurobiology of those with DS to those with ASD [1, 4] or 3) examined the relationship between brain morphology and behavioral presentations (such as cognitive and adaptive skills). This groundbreaking work would be the first to address these gaps in literature.

## Methods

### Participants

The sample consisted of children with DS (*n* = 35), ASD (*n* = 29), and TD (*n* = 80) children. Children included in the analyses were comparable in chronological age. See Table 2 for mean age and age ranges by group.

Children with DS were enrolled based on parent report of trisomy 21 diagnosis and associated phenotypic profiles. The sample with DS was acquired simultaneously with same-aged ASD and typically developing children through the Infant Brain Imaging Study (IBIS), which provided comparison samples in this work. In accordance with the IBIS study design, all ASD children were at high likelihood for autism due to having an older ASD sibling at the time of enrollment (as young as 6 months of age). All children in the ASD group received an ASD diagnosis at their school-age visit associated with this longitudinal study. Children in the TD group were at low likelihood for developing ASD as they did not have an older ASD sibling. Children with an older sibling diagnosed with a known genetic or psychiatric condition were excluded from the TD group, and all TD children had an older TD sibling.

Exclusion criteria across groups included: a diagnosis of a genetic condition or syndrome reported to be associated with autism (with the exception of Down syndrome in the Down syndrome group), a medical or neurological condition known to impact growth and development, significant uncorrected vision or hearing loss (blindness; deafness), low birth weight (< 2000 g) or prematurity (< 36-weeks’ gestation), exposure to in-utero exogenous compounds known to affect the brain adversely (alcohol, certain prescription medications, etc.), non-English speaking families, contraindication for MRI, a family history of intellectual disability, psychosis, schizophrenia, or bipolar disorder in a first degree relative, and adopted subjects.

The IBIS network includes four data collection sites (The University of North Carolina at Chapel Hill, University of Washington, Children’s Hospital of Philadelphia, and Washington University in St. Louis). Parents provided informed consent and children provided assent when appropriate, and the institutional review board at sites approved the research protocol. Note that the children included in this study constitute a subsample from the larger study cohort, chosen based on the availability of processed and usable data at the time this manuscript was written.

### Behavioral assessments

Participants were evaluated with a battery of behavioral and developmental tests. The battery included measures of cognitive ability, social communication, adaptive behaviors, and autism symptoms. Developmental level and adaptive functioning were assessed using the Differential Ability Scales (DAS) [31] and the Vineland Adaptive Behavior Scale (VABS) [32], respectively. The Autism Diagnostic Observation Schedule, Second Edition (ADOS-2) [33] was used to collect autism symptoms. Refer to Table 2 for participant demographics alongside cognitive, adaptive, and autism measures.

#### DAS

The School-Age version of the Differential Ability Scales, Second Edition (DAS-II) [31] was utilized to assess cognitive abilities in children and gleans information about a child across multiple subscales. The DAS-II provides standard scores (mean = 100; SD = 15) for the General Conceptual Ability (GCA) score, and additional domains including, Verbal Ability, Nonverbal Reasoning Ability, and Spatial Ability. The DAS was administered by trained research personnel and was designed for children between the ages of 30 months and 17 years, 11 months and was administered by trained research personnel.

#### Vineland

The Vineland Adaptive Behavior Scales, Third Edition (VABS-3) [32] is a parent interview that gleans information about adaptive functioning in school-aged children across 3 subdomains: Socialization, Daily Living Skills and Communication. The VABS provides standard scores (mean = 100; SD = 15) for each of these domains, as well as the and the Adaptive Behavior Composite (ABC) score. The ABC score offers an overall impression of a child’s general level of adaptive functioning (integrating subdomain standard scores), while highlighting specific strengths and weaknesses in a skill profile is assessed within subdomains. The Motor Skills domain was excluded from analyses because the participant’s age was beyond the age range for which the VABS-3 motor domain is standardly administered. Additionally, this domain is not required or included in the ABC score. All assessors underwent formal training in the administration of the VABS, ensuring consistent and accurate assessment across all participants.

#### ADOS

The Autism Diagnostic Observation Schedule, Second Edition (ADOS-2) [33] is a direct observation measure of autism symptoms. The ADOS-2 is only considered valid when conducted without personal protective equipment (PPE). For this reason, only ADOS administrations conducted without PPE, before the onset of the COVID pandemic, were included.

The ADOS-2 is composed of 5 different modules, of which one is selected based on an individual’s age and language level. For this study, module 1 (no to minimal use of words) was conducted with 5 children (2 ASD, 3 DS), module 2 (phrase speech) was conducted with 16 children (1 ASD, 15 DS), and module 3 (fluent speech) was administered for 84 children (50 TD, 19 ASD, and 15 DS). The ADOS-2 has strict reliability and validity standards; research reliability was obtained by all IBIS staff who conducted the ADOS-2. Reliability was maintained through quarterly cross-site reliability calls. The ADOS-2 yields calibrated severity scores (CSSs), ranging from 1 (least impairment) to 10 (most impairment) across social affect (SA), restricted and repetitive behaviors (RRBs), and overall (CSS); these scores allow for cross module comparisons of autism symptoms. In our group with DS, 11 (31%) met cut-offs for autism spectrum disorder (ASD) on the ADOS-2. However, none of the children with DS were formally diagnosed with ASD based on clinician impressions.

### MRI methods

#### MRI acquisition

MRI scans were performed at each site on identical 3 T Siemens Prisma scanners using a 32-channel head coil. A board-certified behavior analyst (BCBA, author AS) trained site personnel at the beginning of the study and provided ongoing consultation to clinical sites. The BCBA developed an MRI-specific protocol of behavioral training methods based on applied behavior analysis, which demonstrated high success in acquiring scans from school-age ASD children, including those with co-morbid intellectual disability [34] without the need for sedation. This training protocol included an assent-based desensitization strategy, including methods to train participants to lie still in the scanner. In addition to this, the protocol included a desensitization stage where the participants prepared for the scan in a “Mock” MRI scanner (non-functional), introducing the child to the sounds and stimuli associated with the MRI while practicing remaining still. The structural imaging protocol included two scans of 3D T1 weighted MPR: TR = 2500 ms, TE = 2.03 ms, 176 sagittal slices, FOV 240 mm, voxel size = 1.0mm^3^, and *a* 3D T2 weighted SPC: TR = 3200 ms, TE = 564 ms, 176 sagittal slices, FOV 240 mm, voxel size 1.00mm^3^.

#### Structural MRI Quality Control (QC)

Structural MRI data was assessed quantitatively and visually to detect MRI artifacts (e.g., excessive motion, insufficient coverage, or ghosting). Two raters, one for volumetric measurements and one for cortical surface measurements, respectively, visually assessed image quality for the entire study, assigning a QC score and determining usability. This single rater was trained and reliable with other raters in the Neuro Image Research and Analysis Laboratories (NIRAL).

Of the sample that passed initial QC and were used for processing, none failed total cerebral volume (TCV; excluding the brainstem and cerebellum), total tissue, or total GM and WM, but there were some failures in lobar volumes (16 failed for frontal and parietal and 1 for occipital) and 6 for total surface area. Additionally, some volume measurements could not be calculated. Specifically, in the group with DS, 4 children did not have measurements for the left or right frontal and parietal WM and GM volumes. One child in this group did not have measurements for the left and right occipital GM and WM volumes, and surface area measurements. In the ASD group, 3 children did not have left or right frontal and parietal WM volumes.

The total tissue measurement includes GM and WM from the cerebrum, subcortical regions, cerebellum, and the brainstem, which includes the inferior extent of the cerebellum. More details on the procedures for IBIS and scanner QC can be found elsewhere (see [35]).

#### Image processing

Global and lobar brain tissue volumes were derived from each scan that passed QC. All processing was conducted blind to group, sex, and diagnostic information. The T1-weighted (T1w) and T2-weighted (T2w) brain images underwent a correction process for intensity non-uniformity employing the N4 bias field correction algorithm [36] as well as rigid transformation to stereotactic space [37, 38]. To mask the brain, a majority voting approach was employed, which included the joint warping and registration of T1w and T2w images with the best outcome and six predefined atlases, either single or averaged. Additional manual adjustments to the brain masks were made with the itkSNAP software [36] to ensure maximum accuracy.

For the segmentation process, tissues were segmented into whole brain WM and GM followed by a multi-atlas based regional parcellation. The definition of the regions was based on a single-atlas label based ROI template, as well as 18 selected and edited tissue templates from the pilot study (without participants with DS). A multi-modality (T1w and T2w) multi-atlas segmentation workflow was employed, utilizing the in-house, open-source MultiSegPipeline software [39]. This software facilitated the label fusion techniques derived from the Advanced Normalization Tools (ANTs) toolset [40]. An extensive visual assessment was conducted to ascertain the segmentation quality of all images in terms of anatomical accuracy, leading to the conclusion that no processed data warranted exclusion based on the segmentation quality.

The cortical surface area (SA) was derived using a modified CIVET workflow ([41, 42]), which involved the application of tessellate and deformable surface evolution to WM, followed by the expansion of the WM surface to the boundary between GM and CSF. After mapping to spherical domain and co-registration using cortical features such as the sulcal depth [42], the regional SA was then calculated using a simple one-ring neighborhood extraction method. Measurements were taken at the mid-cortical surface, averaging surfaces from the computed white and pial surfaces. Both surfaces underwent visual quality control (QC) with surface cuts overlaid on the magnetic resonance (MR) images for verification.

Table 1 provides a comprehensive list of the brain regions examined in this manuscript, along with a comparison to existing neuroimaging studies on DS (to TD) that report findings related to these regions, if available.

**Table 1:** Comparison of brain regions studied with existing DS neuroimaging literature

Neuroimaging	Existing DS Neuroimaging Literature	
**Total volumes:**	Larger	Smaller
Cerebrum		5, 6, 7, 8, 9, 13
White matter (W)		8, 9, 13
Gray matter (G)		8
Tissue*		
**Cerebral Surface Area**		
**Lobar brain tissues (left and right):**		
Cerebrum ^G,W^		
Frontal ^G,W^		9^W^
Parietal ^G,W^	8, 9^G^; 9^W^	
Occipital ^G,W^	9^G^	
Temporal ^G,W^	9^G^; 8, 9^W^	
Cerebellum ^G,W^		

^*^This measurement includes total G and W volume from the whole cerebrum, which includes subcortical regions, the cerebellum, and the brainstem

### Analyses

Analyses of variance (ANOVAs) were used to compare descriptive, cognitive, and adaptive behaviors across DS, ASD, and TD groups. Analyses of Covariance (ANCOVAs) were conducted to evaluate group differences on brain volume metrics while controlling for total cerebral volume (TCV), age and sex. Age-squared was explored as a confounding variable, but no significant relationships were found; therefore, it was eliminated from analyses. Separate correlations by group were conducted to evaluate the relationship between select brain volumes and cognition (DAS), adaptive behaviors (VABS) and autism symptoms (ADOS). See Table 1 for an overview of which variables were selected. For all analyses, an alpha level of 0.001 was used to account for multiple comparisons [43, 44]. Data analyses and visualization were conducted via SPSS and Matlab [45].

## Results

Children with DS were compared to TD and ASD children to provide a comprehensive understanding of cognitive abilities, adaptive behaviors, global brain volumes and surface area measurements. These analyses explored potential relationships between brain volumes and behavioral outcomes. The following results detail the findings from the ANOVAs, ANCOVAs, and correlations which offer insight into behavioral profiles and differences in brain volumes and surface area across groups.

### Cognitive

Analyses revealed statistically significant differences (TD > ASD > DS) across groups on the DAS GCA, (*p* < 0.001), Verbal (*p* < 0.001), and Spatial (*p* < 0.001) domains, such that TD children scored higher than children with ASD who, in turn, scored higher than children with DS. Additionally, children with DS scored significantly lower (*p* < 0.001) than ASD children in Non-Verbal Reasoning (DS < ASD, TD). See Table 2 for range of scores.

**Table 2:**
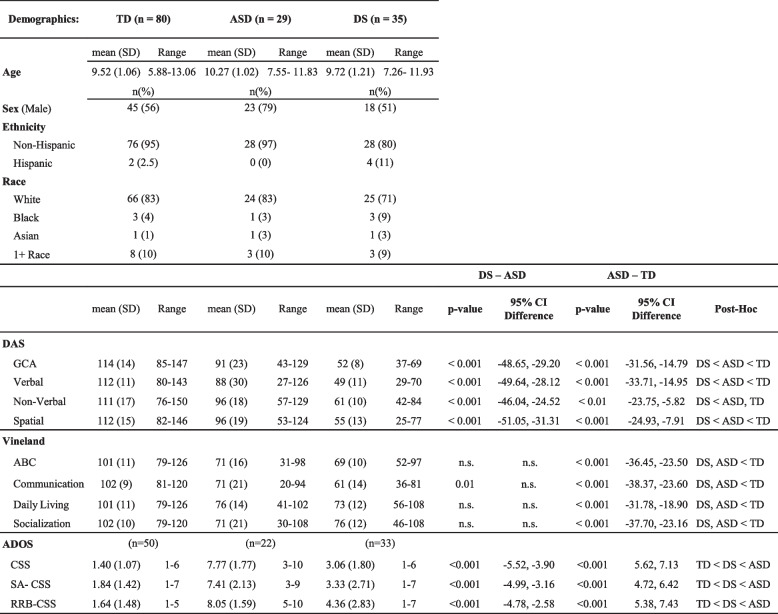
Participant demographics and cognitive, adaptive, autism measures

### Adaptive

Analyses of overall Vineland ABC scores revealed the DS and ASD groups were significantly lower than the TD group (*p* < 0.001, TD > ASD = DS). This relationship was also present in the Communication (*p* < 0.001), Daily Living (*p* < 0.001) and Socialization (*p* < 0.001) domains. See Table 2 for details.

### Autistic symptoms

Analyses of CSS, CSS-SA, and CSS-RRB revealed statistically significant differences (*p* < 0.001) between all groups such that TD group had lower (fewer autism symptoms) scores than the DS group which had lower scores than those with ASD (TD < DS < ASD).

### Global brain volumes and surface area

**Fig. 1:**
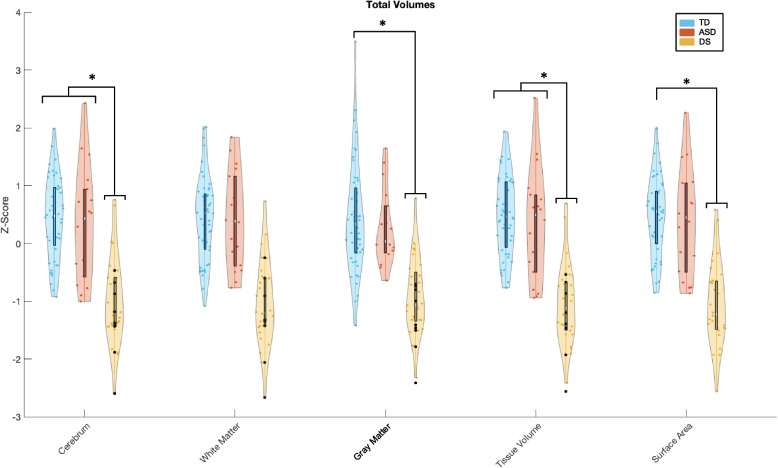
Total volumes and surface area: violin plots by group. *Comparison between DS and other groups: Stars indicate significant differences between the DS group and both ASD and TD groups (*p* < 0.001) except for total surface area (TD > DS) and gray matter (TD > DS)

Age and sex were found to significantly interact with several brain volume metrics, as shown in Table 3. ANCOVA analyses indicated that TCV and total tissue volume had significant associations between all groups. TCV in children with DS was significantly smaller than both the TD and ASD groups (*p* < 0.001 vs. TD, *p* < 0.001 vs. ASD). The same relationship was found in total tissue volume (*p* < 0.001, *p* < 0.001). The total surface area and total GM was significantly smaller in DS compared to TD (*p* < 0.001, *p* < 0.001 respectively), but not significantly different from ASD (*p* = 0.002, *p *= 0.003 respectively). No significant differences were found across groups in total WM volume (*p* = 0.03) in the primary ANCOVA; therefore pairwise analyses were not run. Of note, analyses of specific WM regions indicated significant regional differences across frontal, temporal, and cerebellar WM (see *Lobar and Cerebellar White Matter* section below). See Table 3 and Fig. 1.

**Table 3:**
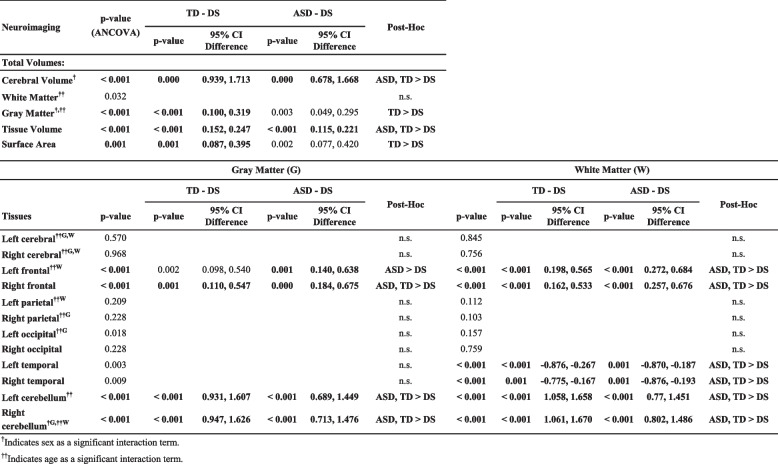
ANCOVAs for brain volumes

### Lobar and cerebellar Gray Matter (GM)

Age and sex had a significant relationship with total GM volume (*p* < 0.001). ANCOVA analyses indicated that GM volumes of the left and right frontal lobe and left and right cerebellum were significantly different across groups. Pairwise tests revealed the DS group had significantly smaller volumes than the ASD group in the left frontal lobe (*p* = 0.001) but not the TD group (*p* = 0.002). However, the DS group had significantly smaller volumes than both ASD and TD groups in the left (*p* < 0.001, *p* < 0.001) and right cerebellum (*p* < 0.001, *p* < 0.001). See Table 3 and Supplementary Fig. 1.

### Lobar and cerebellar White Matter (WM)

ANCOVA results revealed that WM volumes of the left and right frontal lobes, temporal lobes, and cerebellum were different across groups. The DS group had significantly smaller volumes than both the TD and ASD groups in left (*p* < 0.001 vs. TD, *p* < 0.001 vs. ASD) and right frontal lobes (*p* < 0.001, *p* < 0.001), left (*p* < 0.001, *p* < 0.001) and right cerebellum (*p* < 0.001, *p* < 0.001), and left (*p* < 0.001, *p* = 0.001) and right (*p* = 0.001, *p* = 0.001) temporal lobes. Other comparisons were not statistically significant, as detailed in Table 3. See Supplementary Fig. 2.

### Brain-behavior associations

Vineland Daily Living scores were significantly positively associated with left and right GM and WM cerebellum volumes in the DS group only, such that larger GM and WM cerebellum volumes associated with higher or better adaptive skills. No significant correlations were observed with ABC, Socialization, and Communication scores.

In the ASD group, TCV was significantly negatively associated with DAS GCA (*p* < 0.001), meaning that larger TCV was associated with lower or worse global cognitive ability. Similarly, DAS GCA was negatively associated with total GM (*p* < 0.001) and total tissue (*p* < 0.001) volumes in the ASD group. The total surface area was also negatively associated with DAS GCA in the ASD group, indicating that higher GCA scores (better cognitive ability) was associated with smaller surface area volumes (*p* < 0.001).

**Fig. 2:**
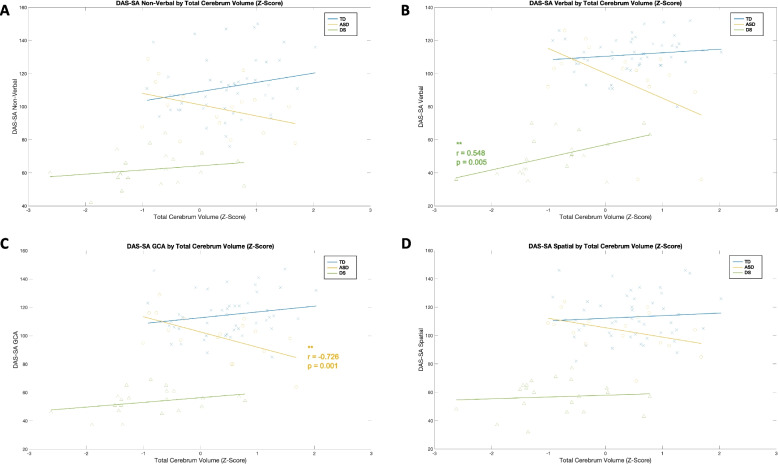
Total cerebral volume and DAS domains

**Fig. 3:**
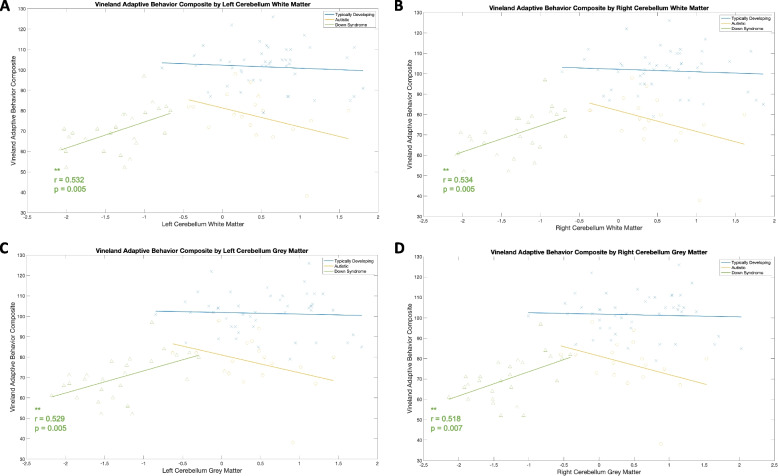
Cerebellar GM and WM z-scores across Vineland ABC scores

No significant correlations between ADOS CSS, CSS-SA, and CSS-RRB and regional brain volumes was observed. Some of these correlations, such as the relationship between CSS-RRB and left cerebellum volumes in the DS group (*p* = 0.02), did not meet our alpha level to deem significant, though trends indicate these relationships should continue to be explored in larger samples. See Figs. 2 and 3 and Supplementary Table 1.

## Discussion

This study characterized selected brain volumes in school-aged children with DS in contrast to children with ASD and those with typical development. We found converging findings with the literature demonstrating that children with DS have lower cognitive and adaptive scores compared to TD children. However, despite having significantly lower cognitive skills, children with DS showed comparable adaptive scores to those with ASD. Contrasting the commonly reported observation of a discrepancy between ability and “performance” in ASD, children with DS demonstrated adaptive skills consistent with their cognitive skills [46–48]. About one-third of children with DS met cutoffs for autism on the ADOS, consistent with published findings [49]. Although none of the children with DS received clinical a diagnosis of autism, these ADOS findings demonstrate that a large proportion of school age children with DS exhibited elevated autistic characteristics. However, these results may overlap with characteristics associated with DS, rather than indicating a clear diagnosis of ASD. This highlights the need for careful interpretation when using the ADOS in DS, as it can affect the specificity of this measure for diagnosing ASD.

To date, neuroimaging studies of DS are limited by small samples and broad age ranges [1, 6, 8, 50–52]. To our knowledge, this study is the first to examine brain volumes in school age children with DS and the first to contrast that to school age children with idiopathic ASD. Consistent with prior reports of other DS groups, we observed that children with DS have significantly smaller brains than TD and the ASD comparison groups [53–55]. A trend towards similar differences was observed in total GM volume, total tissue volume, and total cortical surface area, right frontal GM and WM, left frontal WM, and bilateral cerebellar GM and WM volumes. However, while children with DS were observed to have smaller total brain volume, the differences across brain regions varied by structure. For example, when controlling for differences in TCV, age, and sex, parietal, temporal, or occipital lobe volumes (left and right; GM and WM) did not significantly differ. In contrast, right frontal and cerebellar lobe volumes (left and right; GM and WM) were smaller than comparison groups when controlling for the aforementioned TCV, age, and sex. This is consistent with previously published findings in children with DS that show preserved parietal and temporal lobe GM volume despite smaller overall brain volume than TD counterparts [13, 55]. Further exploration of the structural neuroanatomy of DS is necessary, as studies have been inconsistent with some showing volume reductions of WM in temporal and parietal lobes [13, 56]. The underlying functional implications of disproportionally larger region-specific volumetric decreases is unknown, though could reflect particularly impaired higher order deficits (e.g., frontal-mediated) versus relatively intact somatosensory abilities (e.g., parietal-mediated), though both functions are impaired compared to typical development [57–59]. This hypothesis is consistent with previous research that has shown volume reduction in the frontal lobes of the DS brain [13] and may be related to decreased cognitive and executive functioning in individuals with DS [23].

This study is the first to include an ASD comparison sample and link behavioral and brain volume findings. The reduced brain volumes seen in children with DS has motivated researchers to delve into genetic and neuronal factors contributing to this abnormality. Transcriptomic and induced pluripotent stem cell (iPSC) studies have consistently reported altered gene expression on chromosome 21, albeit to varying degrees [60–64]. Martinez et al. (2024) utilized in vitro DS models with iPSCs and identified consistent genes altered during neural induction, including TIAM1, CHODL, PSCP4, and TTC3 [60]. These genes play roles in neurite growth, neuron differentiation and maturation [60], likely associated with downstream volume differences in DS. Postmortem studies of DS have found decreased synaptic density and length, and fewer dendritic spines which are critical for neuronal communication [65–68]. Collectively, these observations are associated with an overexpression of DYRK1A, APP, and S1OOβ genes located on chromosome 21 [69–71]. The overexpression in DYRK1A and APP genes has been reported to be associated with brain development [69], and disrupting mitochondrial redox states in DS nervous system stem cells, or neural progenitor cells thereby increasing cell death [71]. S1OOβ is involved in growth and function of neurons, specifically playing a role in neuroblasts and glia function [72]. Overexpression of S1OOβ has been linked to dendritic abnormalities, neuroinflammation and neuronal damage [70].

We found no significant difference in occipital lobe tissue volume between DS and comparison groups under the control variables. Most (60–80%) children with DS often have atypical visual function and poorer visual acuity [73], including conditions such as nystagmus (3–33%), strabismus (36%) and others [74]. In addition, impairment in adaptive behavior is associated with larger visual impairment in children with DS [75]. The lack of difference in occipital volumes may indicate that atypical visual function in the DS group is most prominent in lower-order structures (e.g., optic nerve, retina, etc.), but may not fundamentally alter factors that regulate volume (e.g., neurogenesis, dendritic branching, and synapse formation, or pruning mechanisms) in cortical areas that process visual information. While we did not specifically address this relationship due to sample size, future work should continue to explore the relationship between adaptive behaviors, neurobiology, and visual impairment in DS samples.

Other studies have identified a relationship between IQ and global brain volumes such that larger brains are correlated with greater cognitive skills in typical development [76–78]. This has also been reported in individuals with psychiatric conditions, including schizophrenia and Bipolar I Disorder [76, 77]. In this study, there was not an association between cognitive abilities and brain volumes in the TD and DS groups. However, there was a trend that did not reach statistical significance in the DS group that larger brains (TCV) were associated *verbal *cognitive skills. It is possible that a larger sample of children with DS would have revealed a statistically significant relationship. One study of DS has shown an association between cognitive abilities and brain regions, such as positive correlations between amygdala and hippocampal volumes with memory tests [79]. While intriguing, it is important to note that our DS sample had a rather narrow range of cognitive ability, which may limit interpretation of this data.

The ASD group showed a negative relationship between brain volume (TCV) and cognitive skills (GCA score) such that larger TCV was associated with poorer cognitive skills. This negative association was also observed across total tissue volume, and total GM volume. Total WM volumes and total tissue volumes were also negatively trending with spatial scores, specifically, on the DAS, in the ASD group only. This suggests that, unlike the positive association between cognitive skills and brain size found in typical development [76–78], at a certain point, larger brains may be disadvantageous for ASD children, particularly in the spatial domain. Research has suggested that early brain overgrowth is a marker of autism in pre-symptomatic infants and toddlers who go on to receive autism diagnoses, highlighting the potential atypicality of larger brains in this population [35].

This study’s findings should be interpreted considering several limitations. The research employed the Differential Ability Scales (DAS) [80], the Vineland Adaptive Behavior Scales (VABS) [32], and the Autism Diagnostic Observation Schedule (ADOS-2) [33], to characterize the participants behavioral profiles. It should be noted that the validity of these measures has not explicitly been confirmed in DS samples, as studies have reported floor effects [81–83]. It is possible that the values derived from these metrics are poor representations of the constructs that they intend to reflect. Indeed, none of the children with DS in our study received co-diagnoses of autism; yet more than 30% of the DS sample met cutoffs for autism on the ADOS. Meta-analytic reviews indicate that the specificity of the ADOS ranges from 0.81–0.85 [84], using a sample of non-ASD individuals with varied diagnoses, among which only a small subsample had DS. Nevertheless, the specificity of the ADOS is reportedly better in these mixed samples than we see in our DS-only sample.

The individuals included in this sample, particularly those with available neuroimaging data, may not be representative of the broader populations. For example, individuals with higher cognitive ability and functional capacities who can complete an MRI scan while awake and remain still are more likely to be recruited. Specifically, the children with DS with neuroimaging data might represent a subset of children who have higher cognitive skills, fewer hyperactivity symptoms, sensory sensitivities or anxiety related behaviors, than most individuals with DS. Within our neuroimaging data, there is some missing regional information because of participant movement in the scanner. While this data loss is minimal, it is possible that, when using relatively small samples such as ours, these losses of data may affect the power of our sample and subsequent ability to detect differences of significance. This may also be reflected in the ADOS-2 results since we were limited to using data that was not collected during the COVID-19 pandemic, when PPE was in use and invalidated data collection. As such, all results presented here should be replicated in larger samples. Additionally, DS research should strive to include diverse clinical presentations to better understand the range of neurodevelopmental characteristics associated with DS.

DS is a highly underrepresented group in neurobiological studies. This research addresses a gap in the literature comparing the neurobiology of DS to ASD individuals, measured by MRI. Despite the considerable behavioral overlap between these prevalent neurodevelopmental disorders [26], this study represents a crucial initial effort toward understanding the neurobiology of DS using neuroimaging techniques. We reveal differences and similarities in cortical volumes across groups and connect these findings to unique cognitive and adaptive profiles. Our results highlight the unique neurobiological features of DS despite similarities with ASD individuals in behavioral (e.g., adaptive) skills. This study is a steppingstone to future research comparing DS brain and behavioral characteristics to ASD individuals and TD samples, as well as other neurodevelopmental disorders with similar phenotypic presentations.

## Supplementary Information

Supplementary Material 1: Supplementary Fig. 1. Gray Matter: Violin Plots by group.*Comparison between DS and other groups: Stars indicate significant differences between the DS group and both ASD and TD groups (*p* < 0.001), except for left frontal GM (ASD > DS).

Supplementary Material 2: Supplementary Fig. 2. Violin Plots: White Matter by Group. *Comparison between DS and other groups: Stars indicate significant differences between the DS group and both ASD and TD groups (*p* < 0.001).

Supplementary Material 3: Supplementary Table 1. Brain-Behavior Correlations.

## Data Availability

No datasets were generated or analysed during the current study.

## Abbreviations

ABC: Adaptive Behavior Composite
ADOS-2: Autism Diagnostic Observation Scale, Second Edition
ANCOVA: Analyses of Covariance
ANOVA: Analyses of Variance
ASD: Autism spectrum disorder
BCBA: Board-certified behavior analyst
DAS: Differential Ability Scales
DS: Down syndrome
GCA: General Conceptual Ability
GM: Gray matter
IBIS: Infant Brain Imaging Study
ID: Intellectual disability
IQ: Intelligence Quotient
MRI: Magnetic resonance imaging
NDD: Neurodevelopmental disorders
NIRAL: Neuro Image Research and Analysis Laboratories
QC: Quality Control
TCV: Total Cerebral Volume
TD: Typically developing
VABS: Vineland Adaptive Behavior Scales
WM: White matter

## References

[1] Hamner T, Udhnani MD, Osipowicz KZ, Lee NR. Pediatric Brain Development in Down Syndrome: A Field in Its Infancy. J Int Neuropsychol Soc. 2018;24(9):966–76. 10.1017/S1355617718000206. 29789029 10.1017/S1355617718000206 PMC6207466

[2] Parker SE, Mai CT, Canfield MA, et al. Updated National Birth Prevalence estimates for selected birth defects in the United States, 2004–2006. Birt Defects Res A Clin Mol Teratol. 2010;88(12):1008–16. 10.1002/bdra.20735. 10.1002/bdra.20735 20878909

[3] CDC. Facts about Down Syndrome | CDC. Centers for Disease Control and Prevention. Published October 10, 2023. Accessed 22 Feb 2024. https://www.cdc.gov/ncbddd/birthdefects/downsyndrome.html.

[4] Hamadelseed O, Chan MKS, Wong MBF, Skutella T. Distinct neuroanatomical and neuropsychological features of Down syndrome compared to related neurodevelopmental disorders: a systematic review. Front Neurosci. 2023;17:1225228. 10.3389/fnins.2023.1225228. 37600012 10.3389/fnins.2023.1225228 PMC10436105

[5] Kates WR, Folley BS, Lanham DC, Capone GT, Kaufmann WE. Cerebral growth in Fragile X syndrome: review and comparison with Down syndrome. Microsc Res Tech. 2002;57(3):159–67. 10.1002/jemt.10068. 12112452 10.1002/jemt.10068

[6] Smigielska-Kuzia J, Boćkowski L, Sobaniec W, et al. A volumetric magnetic resonance imaging study of brain structures in children with Down syndrome. Neurol Neurochir Pol. 2011;45(4):363–9. 10.1016/s0028-3843(14)60107-9. 22101997 10.1016/s0028-3843(14)60107-9

[7] Pinter JD, Brown WE, Eliez S, Schmitt JE, Capone GT, Reiss AL. Amygdala and hippocampal volumes in children with Down syndrome: a high-resolution MRI study. Neurology. 2001;56(7):972–4. 10.1212/wnl.56.7.972. 11294940 10.1212/wnl.56.7.972

[8] Pinter JD, Eliez S, Schmitt JE, Capone GT, Reiss AL. Neuroanatomy of Down’s syndrome: A high-resolution MRI study. Am J Psychiatry. 2001;158(10). 10.1176/appi.ajp.158.10.1659. 10.1176/appi.ajp.158.10.1659 11578999

[9] Lee NR, Adeyemi EI, Lin A, et al. Dissociations in Cortical Morphometry in Youth with Down Syndrome: Evidence for Reduced Surface Area but Increased Thickness. Cereb Cortex N Y N 1991. 2016;26(7):2982–90. 10.1093/cercor/bhv107. 10.1093/cercor/bhv107 PMC4898663 26088974

[10] Gunbey HP, Bilgici MC, Aslan K, et al. Structural brain alterations of Down’s syndrome in early childhood evaluation by DTI and volumetric analyses. Eur Radiol. 2017;27(7). 10.1007/s00330-016-4626-6. 10.1007/s00330-016-4626-6 27798752

[11] Powell D, Caban-Holt A, Jicha G, et al. Frontal white matter integrity in adults with Down syndrome with and without dementia. Neurobiol Aging. 2014;35(7). 10.1016/j.neurobiolaging.2014.01.137. 10.1016/j.neurobiolaging.2014.01.137 PMC3992921 24582640

[12] Romano A, Moraschi M, Cornia R, et al. White matter involvement in young non-demented Down’s syndrome subjects: a tract-based spatial statistic analysis. Neuroradiology. 2018;60(12):1335–41. 10.1007/s00234-018-2102-5. 30264168 10.1007/s00234-018-2102-5

[13] Carducci F, Onorati P, Condoluci C, et al. Whole-brain voxel-based morphometry study of children and adolescents with Down syndrome. Funct Neurol. 2013;28(1). 10.11138/FNeur/2013.28.1.019. PMC3812718 23731912

[14] Menghini D, Costanzo F, Vicari S. Relationship between brain and cognitive processes in Down syndrome. Behav Genet. 2011;41(3):381–93. 10.1007/s10519-011-9448-3. 21279430 10.1007/s10519-011-9448-3

[15] Dierssen M, Vallina IF, Baamonde C, et al. Impaired cyclic AMP production in the hippocampus of a Down syndrome murine model. Brain Res Dev Brain Res. 1996;95(1):122–4. 10.1016/0165-3806(96)00071-5. 8873983 10.1016/0165-3806(96)00071-5

[16] Kleschevnikov AM, Belichenko PV, Villar AJ, Epstein CJ, Malenka RC, Mobley WC. Hippocampal long-term potentiation suppressed by increased inhibition in the Ts65Dn mouse, a genetic model of Down syndrome. J Neurosci Off J Soc Neurosci. 2004;24(37):8153–60. 10.1523/JNEUROSCI.1766-04.2004. 10.1523/JNEUROSCI.1766-04.2004 PMC6729789 15371516

[17] Harashima C, Jacobowitz DM, Witta J, et al. Abnormal Expression of the GIRK2 Potassium Channel in Hippocampus, Frontal Cortex and Substantia Nigra of Ts65Dn Mouse: A Model of Down Syndrome. J Comp Neurol. 2006;494(5):815–33. 10.1002/cne.20844. 16374808 10.1002/cne.20844 PMC2929960

[18] García-Cerro S, Vidal V, Lantigua S, et al. Cerebellar alterations in a model of Down syndrome: The role of the Dyrk1A gene. Neurobiol Dis. 2018;110:206–17. 10.1016/j.nbd.2017.12.002. 29221819 10.1016/j.nbd.2017.12.002

[19] Altafaj X, Ortiz-Abalia J, Fernández M, et al. Increased NR2A expression and prolonged decay of NMDA-induced calcium transient in cerebellum of TgDyrk1A mice, a mouse model of Down syndrome. Neurobiol Dis. 2008;32(3):377–84. 10.1016/j.nbd.2008.07.024. 18773961 10.1016/j.nbd.2008.07.024

[20] Pennington BF, Moon J, Edgin J, Stedron J, Nadel L. The neuropsychology of Down syndrome: evidence for hippocampal dysfunction. Child Dev. 2003;74(1):75–93. 10.1111/1467-8624.00522. 12625437 10.1111/1467-8624.00522

[21] Diniz NLF, Parlato-Oliveira E, Pimenta PGA, de Araújo LA, Valadares ER. Autism and Down syndrome: early identification and diagnosis. Arq Neuropsiquiatr. 2022;80(6):620–30. 10.1590/0004-282X-ANP-2021-0156. 35946706 10.1590/0004-282X-ANP-2021-0156 PMC9387185

[22] Lowenthal R, Paula CS, Schwartzman JS, Brunoni D, Mercadante MT. Prevalence of pervasive developmental disorder in Down’s syndrome. J Autism Dev Disord. 2007;37(7):1394–5. 10.1007/s10803-007-0374-4. 17410415 10.1007/s10803-007-0374-4

[23] Grieco J, Pulsifer M, Seligsohn K, Skotko B, Schwartz A. Down syndrome: Cognitive and behavioral functioning across the lifespan. Am J Med Genet C Semin Med Genet. 2015;169(2). 10.1002/ajmg.c.31439. 10.1002/ajmg.c.31439 25989505

[24] Maenner MJ, Shaw KA, Baio J, et al. Prevalence of autism spectrum disorder among children aged 8 Years-Autism and developmental disabilities monitoring network, 11 Sites, United States, 2016. MMWR Surveill Summ. 2020;69(4). 10.15585/MMWR.SS6904A1. 10.15585/mmwr.ss6904a1 PMC7119644 32214087

[25] Barbaro J, Dissanayake C. Developmental profiles of infants and toddlers with Autism Spectrum Disorders identified prospectively in a community-based setting. J Autism Dev Disord. 2012;42(9). 10.1007/s10803-012-1441-z. 10.1007/s10803-012-1441-z 22310906

[26] Bradbury KR, Anderberg EI, Huang-Storms L, Vasile I, Greene RK, Duvall SW. Co-occurring Down Syndrome and Autism Spectrum Disorder: Cognitive, Adaptive, and Behavioral Characteristics. J Autism Dev Disord. 2021. 10.1007/s10803-021-05016-6. Published online 2021. 10.1007/s10803-021-05016-6 33905067

[27] Bruni M, Cameron D, Dua S, Noy S. Reported Sensory Processing of Children with Down Syndrome. Phys Occup Ther Pediatr. 2010;30(4):280–93. 10.3109/01942638.2010.486962. 20735195 10.3109/01942638.2010.486962

[28] Jatkar A, Garrido D, Zheng S, et al. Toddlers at Elevated Likelihood for Autism: Exploring Sensory and Language Treatment Predictors. J Early Interv. 2023;45(1):39–62. 10.1177/10538151211067227. 36969559 10.1177/10538151211067227 PMC10038203

[29] Mary Lashno. Sensory Integration: Observations of Children with Down Syndrome and Autistic Spectrum Disorders. Published October 1999. Accessed 10 Oct 2022. https://www.kennedykrieger.org/stories/sensory-integration-observations-children-down-syndrome-and-autistic-spectrum-disorders.

[30] Wuang YP, Su CY, Su JH. Wisconsin Card Sorting Test performance in children with developmental coordination disorder. Res Dev Disabil. 2011;32(5):1669–76. 10.1016/j.ridd.2011.02.021. 21458225 10.1016/j.ridd.2011.02.021

[31] Elliott CD, Hale JB, Fiorello CA, Dorvil C, Moldovan J. Differential Ability Scales-II prediction of reading performance: Global scores are not enough. Psychol Sch. 2010;47(7). 10.1002/pits.20499.

[32] Sparrow SS, Cicchetti DV, Balla DA. Vineland Adaptive Behavior Scales, Second Edition, survey interview form/caregiver rating form. Livonia, MN: Pearson Assessments; 2005.

[33] Lord C, Rutter M, DiLavore P, Risi S, Gotham K, Bishop S. Autism Diagnostic Observation Schedule (ADOS-2), Second Edition. Torrance, CA: Western Psychological Corporation; 2012.

[34] Nordahl CW, Mello M, Shen AM, et al. Methods for acquiring MRI data in children with autism spectrum disorder and intellectual impairment without the use of sedation. J Neurodev Disord. 2016;8:20. 10.1186/s11689-016-9154-9. 27158271 10.1186/s11689-016-9154-9 PMC4858915

[35] Hazlett HC, Gu H, Munsell BC, et al. Early brain development in infants at high risk for autism spectrum disorder. Nature. Published online 2017. 10.1038/nature21369. 10.1038/nature21369 PMC5336143 28202961

[36] Tustison NJ, Avants BB, Cook PA, et al. N4ITK: improved N3 bias correction. IEEE Trans Med Imaging. 2010;29(6):1310–20. 10.1109/TMI.2010.2046908. 20378467 10.1109/TMI.2010.2046908 PMC3071855

[37] Fonov VS, Janke A, Caramanos Z, et al. Improved Precision in the Measurement of Longitudinal Global and Regional Volumetric Changes via a Novel MRI Gradient Distortion Characterization and Correction Technique. In: Liao H, Edwards PJ “Eddie,” Pan X, Fan Y, Yang GZ, eds. Medical Imaging and Augmented Reality. Springer; 2010:324–333. 10.1007/978-3-642-15699-1_34.

[38] Fonov V, Evans AC, Botteron K, et al. Unbiased average age-appropriate atlases for pediatric studies. Neuroimage. 2011;54(1):313–27. 10.1016/j.neuroimage.2010.07.033. 20656036 10.1016/j.neuroimage.2010.07.033 PMC2962759

[39] Cherel M, Budin F, Prastawa M, et al. Automatic Tissue Segmentation of Neonate Brain MR Images with Subject-specific Atlases. Proc SPIE-- Int Soc Opt Eng. 2015;9413. 10.1117/12.2082209. 10.1117/12.2082209 PMC4469197 26089584

[40] Tustison NJ, Cook PA, Holbrook AJ, et al. The ANTsX ecosystem for quantitative biological and medical imaging. Sci Rep. 2021;11(1):9068. 10.1038/s41598-021-87564-6. 33907199 10.1038/s41598-021-87564-6 PMC8079440

[41] Kim JS, Singh V, Lee JK, et al. Automated 3-D extraction and evaluation of the inner and outer cortical surfaces using a Laplacian map and partial volume effect classification. Neuroimage. 2005;27(1):210–21. 10.1016/j.neuroimage.2005.03.036. 15896981 10.1016/j.neuroimage.2005.03.036

[42] Ad-Dab’bagh Y, Einarson D, Lyttelton O, et al. The CIVET Image-Processing Environment: A Fully Automated Comprehensive Pipeline for Anatomical Neuroimaging Research. Florence, Italy: Proceedings of the 12th Annual Meeting of the Organization for Human Brain Mapping; 2006.

[43] Chen SY, Feng Z, Yi X. A general introduction to adjustment for multiple comparisons. J Thorac Dis. 2017;9(6):1725–9. 10.21037/jtd.2017.05.34. 28740688 10.21037/jtd.2017.05.34 PMC5506159

[44] Lee S, Lee DK. What is the proper way to apply the multiple comparison test? Korean J Anesthesiol. 2018;71(5):353–60. 10.4097/kja.d.18.00242. 30157585 10.4097/kja.d.18.00242 PMC6193594

[45] Hoffmann H. violin.m - Simple violin plot using MATLAB default kernel density estimation. Published online 2015.

[46] Bourreau Y, Roux S, Gomot M, Bonnet-Brilhault F, Barthélémy C. Validation of the repetitive and restricted behaviour scale in autism spectrum disorders. Eur Child Adolesc Psychiatry. 2009;18(11):675–82. 10.1007/s00787-009-0028-5. 19452196 10.1007/s00787-009-0028-5

[47] Charman T, Pickles A, Simonoff E, Chandler S, Loucas T, Baird G. IQ in children with autism spectrum disorders: data from the Special Needs and Autism Project (SNAP). Psychol Med. 2011;41(3):619–27. 10.1017/S0033291710000991. 21272389 10.1017/S0033291710000991

[48] Klin A, Saulnier CA, Sparrow SS, Cicchetti DV, Volkmar FR, Lord C. Social and communication abilities and disabilities in higher functioning individuals with autism spectrum disorders: the Vineland and the ADOS. J Autism Dev Disord. 2007;37(4):748–59. 10.1007/s10803-006-0229-4. 17146708 10.1007/s10803-006-0229-4

[49] Dimachkie Nunnally A, Nguyen V, Anglo C, et al. Symptoms of Autism Spectrum Disorder in Individuals with Down Syndrome. Brain Sci. 2021;11(10):1278. 10.3390/brainsci11101278. 34679343 10.3390/brainsci11101278 PMC8533848

[50] Koenig KA, Oh SH, Stasko MR, et al. High resolution structural and functional MRI of the hippocampus in young adults with Down syndrome. Brain Commun. 2021;3(2):fcab088. 10.1093/braincomms/fcab088. 33977271 10.1093/braincomms/fcab088 PMC8100000

[51] McCann B, Levman J, Baumer N, et al. Structural magnetic resonance imaging demonstrates volumetric brain abnormalities in down syndrome: Newborns to young adults. NeuroImage Clin. 2021;32:102815. 10.1016/j.nicl.2021.102815. 34520978 10.1016/j.nicl.2021.102815 PMC8441087

[52] Raschle N, Zuk J, Ortiz-Mantilla S, et al. Pediatric neuroimaging in early childhood and infancy: challenges and practical guidelines. Ann N Y Acad Sci. 2012;1252:43–50. 10.1111/j.1749-6632.2012.06457.x. 22524338 10.1111/j.1749-6632.2012.06457.x PMC3499030

[53] Aylward EH, Habbak R, Warren AC, et al. Cerebellar volume in adults with Down syndrome. Arch Neurol. 1997;54(2):209–12. 10.1001/archneur.1997.00550140077016. 9041863 10.1001/archneur.1997.00550140077016

[54] Jernigan TL, Bellugi U, Sowell E, Doherty S, Hesselink JR. Cerebral morphologic distinctions between Williams and Down syndromes. Arch Neurol. 1993;50(2):186–91. 10.1001/archneur.1993.00540020062019. 8431138 10.1001/archneur.1993.00540020062019

[55] Pinter JD, Eliez S, Schmitt JE, Capone GT, Reiss AL. Neuroanatomy of Down’s syndrome: a high-resolution MRI study. Am J Psychiatry. 2001;158(10):1659–65. 10.1176/appi.ajp.158.10.1659. 11578999 10.1176/appi.ajp.158.10.1659

[56] Raz N, Torres IJ, Briggs SD, et al. Selective neuroanatomic abnormalities in down’s syndrome and their cognitive correlates: Evidence from mri morphometry. Neurology. 1995;45(2). 10.1212/WNL.45.2.356. 10.1212/wnl.45.2.356 7854539

[57] Ackerman S. Major Structures and Functions of the Brain. In: Discovering the Brain. National Academies Press (US); 1992. Accessed 27 Feb 2024. https://www.ncbi.nlm.nih.gov/books/NBK234157/.

[58] Genon S, Reid A, Langner R, Amunts K, Eickhoff SB. How to Characterize the Function of a Brain Region. Trends Cogn Sci. 2018;22(4):350–64. 10.1016/j.tics.2018.01.010. 29501326 10.1016/j.tics.2018.01.010 PMC7978486

[59] Maldonado KA, Alsayouri K. Physiology, Brain. In: StatPearls. StatPearls Publishing; 2024. Accessed 27 Feb 2024. http://www.ncbi.nlm.nih.gov/books/NBK551718/. 31869182

[60] Martinez JL, Piciw JG, Crockett M, et al. Transcriptional consequences of trisomy 21 on neural induction. Front Cell Neurosci. 2024;18. 10.3389/fncel.2024.1341141. 10.3389/fncel.2024.1341141 PMC10865501 38357436

[61] Olmos-Serrano JL, Kang HJ, Tyler WA, et al. Down Syndrome Developmental Brain Transcriptome Reveals Defective Oligodendrocyte Differentiation and Myelination. Neuron. 2016;89(6):1208–22. 10.1016/j.neuron.2016.01.042. 26924435 10.1016/j.neuron.2016.01.042 PMC4795969

[62] Sobol M, Klar J, Laan L, et al. Transcriptome and Proteome Profiling of Neural Induced Pluripotent Stem Cells from Individuals with Down Syndrome Disclose Dynamic Dysregulations of Key Pathways and Cellular Functions. Mol Neurobiol. 2019;56(10):7113–27. 10.1007/s12035-019-1585-3. 30989628 10.1007/s12035-019-1585-3 PMC6728280

[63] Li Z, Klein JA, Rampam S, et al. Asynchronous excitatory neuron development in an isogenic cortical spheroid model of Down syndrome. Front Neurosci. 2022;16:932384. 10.3389/fnins.2022.932384. 36161168 10.3389/fnins.2022.932384 PMC9504873

[64] Meharena HS, Marco A, Dileep V, et al. Down-syndrome-induced senescence disrupts the nuclear architecture of neural progenitors. Cell Stem Cell. 2022;29(1):116-130.e7. 10.1016/j.stem.2021.12.002. 34995493 10.1016/j.stem.2021.12.002 PMC8805993

[65] Baburamani AA, Patkee PA, Arichi T, Rutherford MA. New approaches to studying early brain development in Down syndrome. Dev Med Child Neurol. 2019;61(8):867–79. 10.1111/dmcn.14260. 31102269 10.1111/dmcn.14260 PMC6618001

[66] Schmidt-Sidor B, Wisniewski KE, Shepard TH, Sersen EA. Brain growth in Down syndrome subjects 15 to 22 weeks of gestational age and birth to 60 months. Clin Neuropathol. 1990;9(4):181–90. 2146054

[67] Takashima S, Becker LE, Armstrong DL, Chan F. Abnormal neuronal development in the visual cortex of the human fetus and infant with down’s syndrome. A quantitative and qualitative Golgi study. Brain Res. 1981;225(1):1–21. 10.1016/0006-8993(81)90314-0. 6457667 10.1016/0006-8993(81)90314-0

[68] Wisniewski KE. Down syndrome children often have brain with maturation delay, retardation of growth, and cortical dysgenesis. Am J Med Genet Suppl. 1990;7:274–81. 10.1002/ajmg.1320370755. 2149962 10.1002/ajmg.1320370755

[69] Guedj F, Pereira PL, Najas S, et al. DYRK1A: a master regulatory protein controlling brain growth. Neurobiol Dis. 2012;46(1):190–203. 10.1016/j.nbd.2012.01.007. 22293606 10.1016/j.nbd.2012.01.007

[70] Engidawork E, Lubec G. Molecular changes in fetal Down syndrome brain. J Neurochem. 2003;84(5):895–904. 10.1046/j.1471-4159.2003.01614.x. 12603815 10.1046/j.1471-4159.2003.01614.x

[71] Lu J, Esposito G, Scuderi C, et al. S100B and APP Promote a Gliocentric Shift and Impaired Neurogenesis in Down Syndrome Neural Progenitors. PLoS One. 2011;6(7):e22126. 10.1371/journal.pone.0022126. 21779383 10.1371/journal.pone.0022126 PMC3133657

[72] Jacobs BL, Azmitia EC. Structure and function of the brain serotonin system. Physiol Rev. 1992;72(1):165–229. 10.1152/physrev.1992.72.1.165. 1731370 10.1152/physrev.1992.72.1.165

[73] Zahidi AAA, McIlreavy L, Erichsen JT, Woodhouse JM. Visual and Refractive Status of Children with Down’s Syndrome and Nystagmus. Invest Ophthalmol Vis Sci. 2022;63(2). 10.1167/IOVS.63.2.28. 10.1167/iovs.63.2.28 PMC8883157 35195683

[74] Bull MJ, Trotter T, Santoro SL, et al. Health Supervision for Children and Adolescents With Down Syndrome. Pediatrics. 2022;149(5):e2022057010. 10.1542/peds.2022-057010. 35490285 10.1542/peds.2022-057010

[75] de Weger C, Boonstra FN, Goossens J. Differences between children with Down syndrome and typically developing children in adaptive behaviour, executive functions and visual acuity. Sci Rep. 2021;11(1). 10.1038/s41598-021-85037-4. 10.1038/s41598-021-85037-4 PMC8027651 33828124

[76] Jensen MH, Bak N, Rostrup E, et al. The impact of schizophrenia and intelligence on the relationship between age and brain volume. Schizophr Res Cogn. 2019;15. 10.1016/j.scog.2018.09.002. 10.1016/j.scog.2018.09.002 PMC6176038 30302317

[77] Vreeker A, Abramovic L, Boks MPM, et al. The relationship between brain volumes and intelligence in bipolar disorder. J Affect Disord. 2017;223. 10.1016/j.jad.2017.07.009. 10.1016/j.jad.2017.07.009 PMC5588867 28728036

[78] Bajaj S, Raikes A, Smith R, et al. The Relationship Between General Intelligence and Cortical Structure in Healthy Individuals. Neuroscience. 2018;388. 10.1016/j.neuroscience.2018.07.008. 10.1016/j.neuroscience.2018.07.008 30012372

[79] Krasuski JS, Alexander GE, Horwitz B, Rapoport SI, Schapiro MB. Relation of medial temporal lobe volumes to age and memory function in nondemented adults with Down’s syndrome: implications for the prodromal phase of Alzheimer’s disease. Am J Psychiatry. 2002;159(1):74–81. 10.1176/appi.ajp.159.1.74. 11772693 10.1176/appi.ajp.159.1.74

[80] Elliot CD. Differential Ability Scales-II (DAS-II). Harcourt Assessment; 2007. Accessed 23 Feb 2024. https://scholar.google.com/scholar_lookup?title=Differential+Ability+Scales+(DAS+-+II)&author=C+Elliot&publication_year=1990&#d=gs_cit&t=1708727451036&u=%2Fscholar%3Fq%3Dinfo%3AI0qnosvKrZEJ%3Ascholar.google.com%2F%26output%3Dcite%26scirp%3D0%26hl%3Den.

[81] Esbensen AJ, Hooper SR, Fidler D, et al. Outcome Measures for Clinical Trials in Down Syndrome. Am J Intellect Dev Disabil. 2017;122(3):247–81. 10.1352/1944-7558-122.3.247. 28452584 10.1352/1944-7558-122.3.247 PMC5424621

[82] Schworer EK, Esbensen AJ, Fidler DJ, Beebe DW, Carle A, Wiley S. Evaluating working memory outcome measures for children with Down syndrome. J Intellect Disabil Res. 2022;66(1–2):195–211. 10.1111/jir.12833. 33763953 10.1111/jir.12833 PMC8463631

[83] Schworer EK, Voth K, Hoffman EK, Esbensen AJ. Short-term memory outcome measures: Psychometric evaluation and performance in youth with Down syndrome. Res Dev Disabil. 2022;120:104147. 10.1016/j.ridd.2021.104147. 34922089 10.1016/j.ridd.2021.104147 PMC8724458

[84] Lebersfeld JB, Swanson M, Clesi CD, O’Kelley SE. Systematic Review and Meta-Analysis of the Clinical Utility of the ADOS-2 and the ADI-R in Diagnosing Autism Spectrum Disorders in Children. J Autism Dev Disord. 2021;51(11):4101–14. 10.1007/s10803-020-04839-z. 33475930 10.1007/s10803-020-04839-z

