# Supplementary figures and images for: Brain volumes, cognitive, and adaptive skills in school-age children with Down syndrome
Source: J Neurodev Disord. 2024 Dec 19;16:70. doi: 10.1186/s11689-024-09581-6 (PMC11660842; doi:10.1186/s11689-024-09581-6)

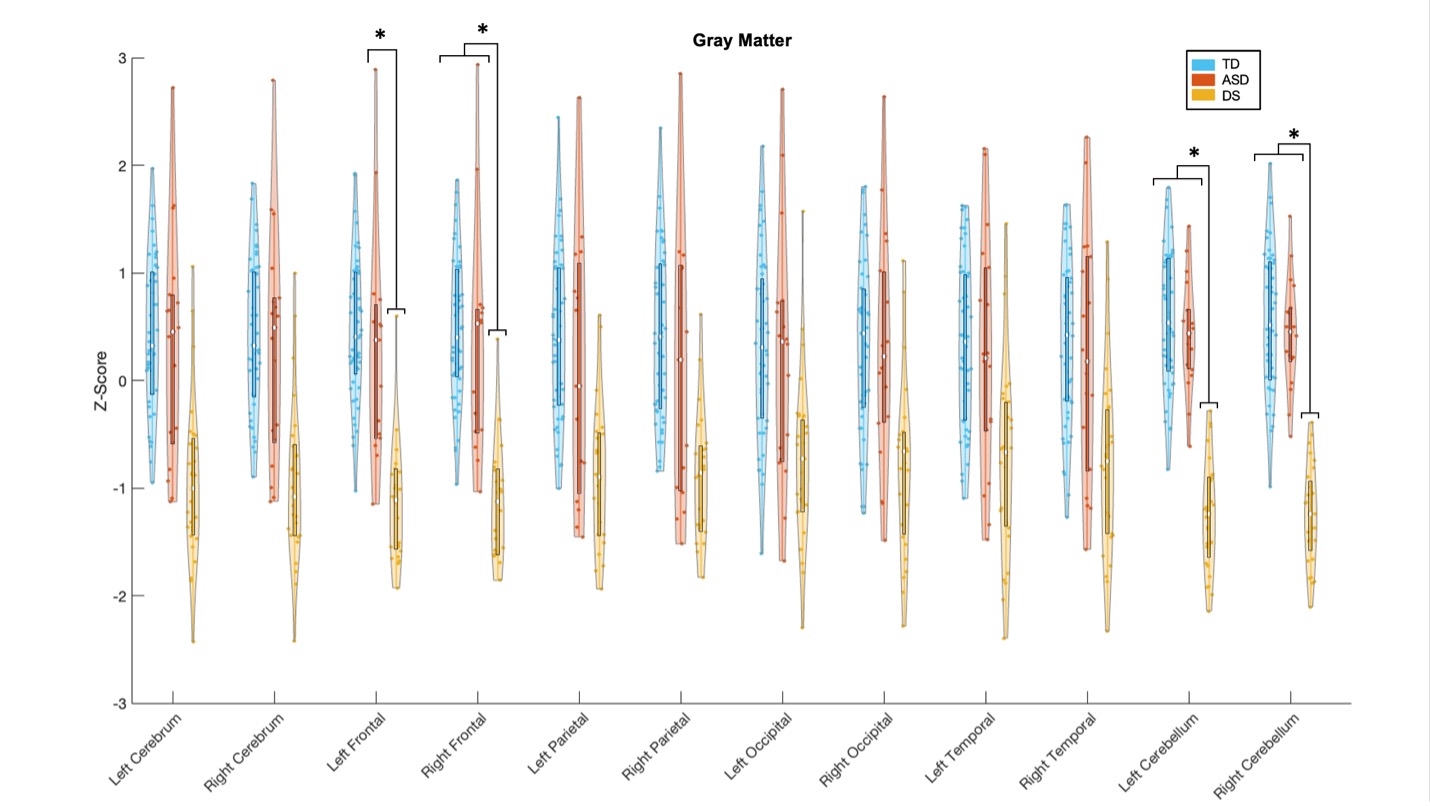

Supplement: Supplementary file 1 — Supplementary Material 1: Supplementary Fig. 1. Gray Matter: Violin Plots by group.*Comparison between DS and other groups: Stars indicate significant differences between the DS group and both ASD and TD groups (p < 0.001), except for left frontal GM (ASD > DS). [file 11689_2024_9581_MOESM1_ESM.jpg]

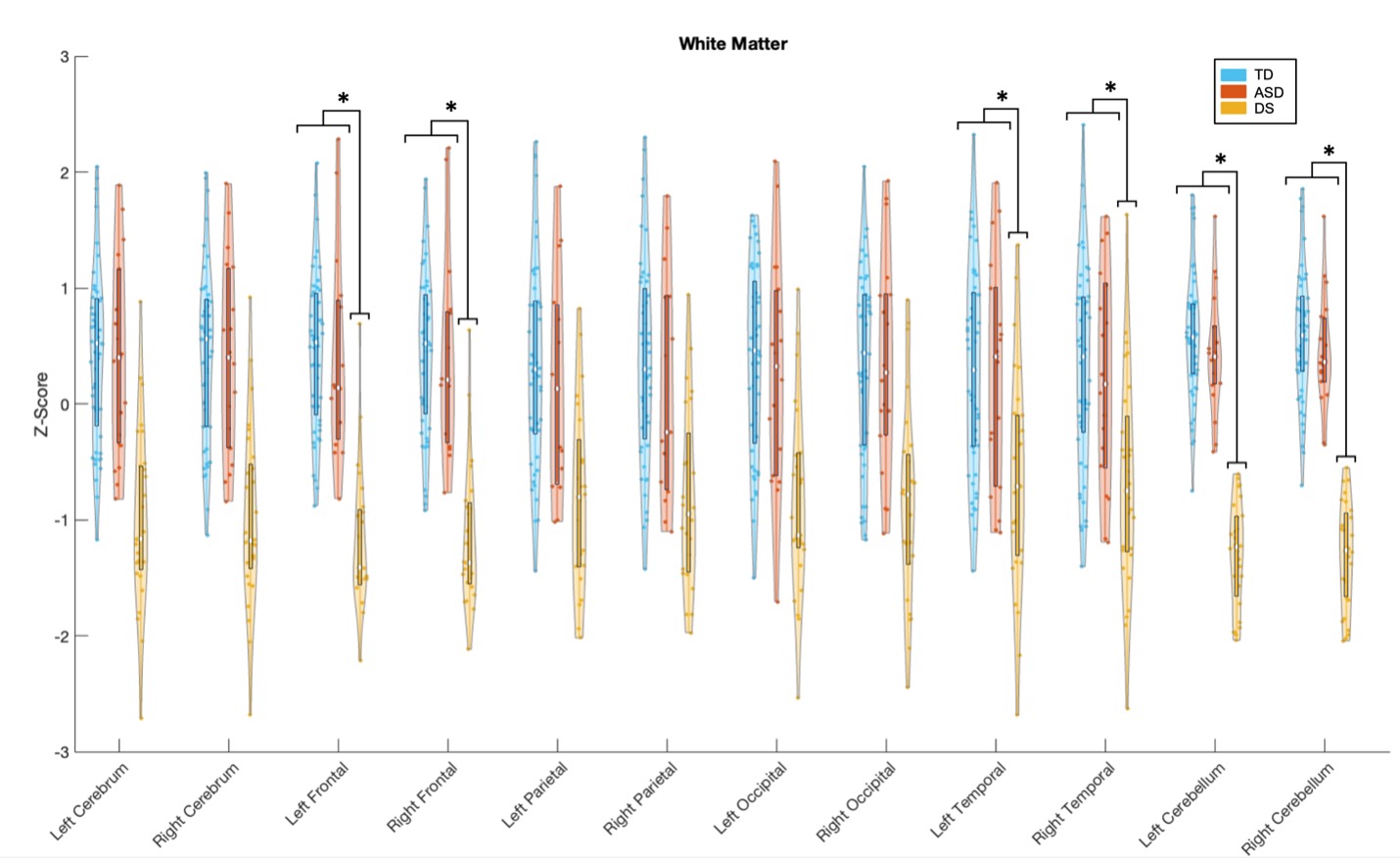

Supplement: Supplementary file 2 — Supplementary Material 2: Supplementary Fig. 2. Violin Plots: White Matter by Group. *Comparison between DS and other groups: Stars indicate significant differences between the DS group and both ASD and TD groups (p < 0.001). [file 11689_2024_9581_MOESM2_ESM.jpg]

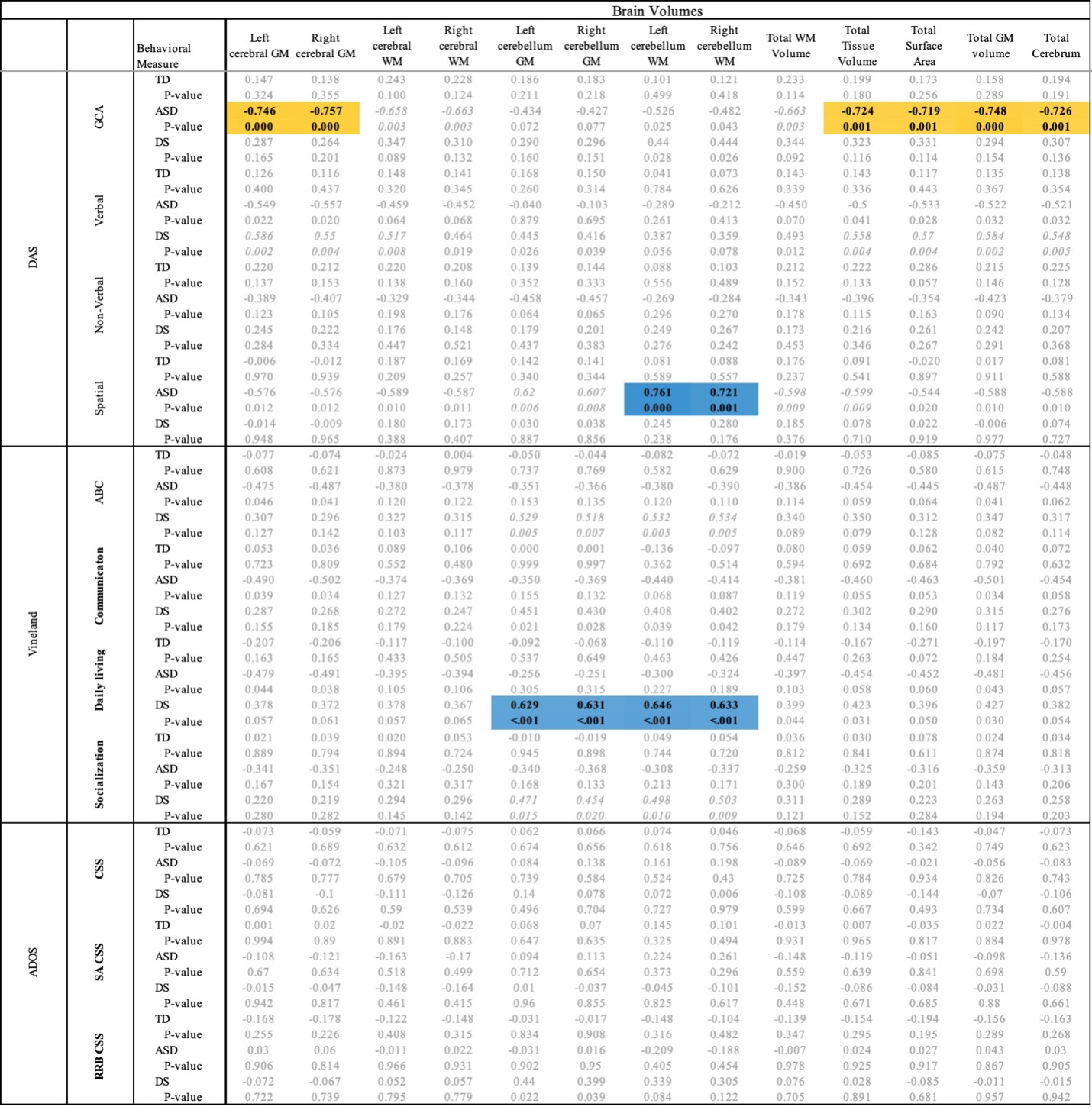

Supplement: Supplementary file 3 — Supplementary Material 3: Supplementary Table 1. Brain-Behavior Correlations. [file 11689_2024_9581_MOESM3_ESM.jpg]
